# Differential effects of environment on potato phenylpropanoid and carotenoid expression

**DOI:** 10.1186/1471-2229-12-39

**Published:** 2012-03-20

**Authors:** Raja S Payyavula, Duroy A Navarre, Joseph C Kuhl, Alberto Pantoja, Syamkumar S Pillai

**Affiliations:** 1 Irrigated Agricultural Research and Extension Center, Washington State University, Prosser, WA 99350, USA; 2 USDA-Agricultural Research Service, Washington State University, 24106 N. Bunn Rd, Prosser, WA 99350, USA; 3 The Department of Plant, Soil, and Entomological Sciences, University of Idaho, P.O. Box 442339, Moscow, ID 84844, USA; 4 USDA- Agricultural Research Service, Subarctic Agricultural Research Unit, P.O. Box 757200, Fairbanks, AK, USA

**Keywords:** phenolics, chlorogenic acid, anthocyanins, carotenoids, gene expression, PAL, antioxidants, potatoes, sucrose, promoters.

## Abstract

**Background:**

Plant secondary metabolites, including phenylpropanoids and carotenoids, are stress inducible, have important roles in potato physiology and influence the nutritional value of potatoes. The type and magnitude of environmental effects on tuber phytonutrients is unclear, especially under modern agricultural management that minimizes stress. Understanding factors that influence tuber secondary metabolism could facilitate production of more nutritious crops. Metabolite pools of over forty tuber phenylpropanoids and carotenoids, along with the expression of twenty structural genes, were measured in high-phenylpropanoid purple potatoes grown in environmentally diverse locations in North America (Alaska, Texas and Florida).

**Results:**

Phenylpropanoids, including chlorogenic acid (CGA), were higher in samples from the northern latitudes, as was the expression of phenylpropanoid genes including phenylalanine ammonia lyase (*PAL*), which had over a ten-fold difference in relative abundance. Phenylpropanoid gene expression appeared coordinately regulated and was well correlated with metabolite pools, except for hydroxycinnamoyl-CoA:quinatehydroxcinnamoyl transferase (*HQT*; r = -0.24). *In silico *promoter analysis identified two cis-acting elements in the HQT promoter not found in the other phenylpropanoid genes. Anthocyanins were more abundant in Alaskan samples and correlated with flavonoid genes including *DFR *(r = 0.91), *UFGT *(r = 0.94) and *F3H *(r = 0.77). The most abundant anthocyanin was petunidin-3-coum-rutinoside-5-glu, which ranged from 4.7 mg g^-1 ^in Alaska to 2.3 mg g^-1 ^in Texas. Positive correlations between tuber sucrose and anthocyanins (r = 0.85), suggested a stimulatory effect of sucrose. Smaller variation was observed in total carotenoids, but marked differences occurred in individual carotenoids, which had over a ten-fold range. Violaxanthin, lutein or zeaxanthin were the predominant carotenoids in tubers from Alaska, Texas and Florida respectively. Unlike in the phenylpropanoid pathway, poor correlations occurred between carotenoid transcripts and metabolites.

**Conclusion:**

Analysis of tuber secondary metabolism showed interesting relationships among different metabolites in response to collective environmental influences, even under conditions that minimize stress. The variation in metabolites shows the considerable phenotypical plasticity possible with tuber secondary metabolism and raises questions about to what extent these pathways can be stimulated by environmental cues in a manner that optimizes tuber phytonutrient content while protecting yields. The differences in secondary metabolites may be sufficient to affect nutritional quality.

## Background

Potatoes (*Solanum tuberosum *L.) are the most consumed vegetable in the United States, and as a staple food are important dietary contributors of phytonutrients. Although they contain only modest amounts of phenylpropanoids, white potatoes are estimated to be the third largest contributor of phenylpropanoids in the American diet [1]. This highlights the importance of staple crops, in which even modest changes in the phytonutrient content can be dietarily significant to an extent not possible in foods consumed in lesser quantities. Secondary metabolites are well known to be subject to environmental control in plants [2]; however, much remains unknown about environmental effects on tuber secondary metabolites, especially in non-extreme conditions where stresses such as drought or severe disease have not been deliberately or inadvertently introduced.

Phenylalanine ammonia lyase (PAL) regulates entry into the phenylpropanoid pathway and is responsive to environmental stimuli including light, pathogens, cold and heat stress [3-6]. Similar effects occur on compounds downstream in the pathway including anthocyanins, which are induced by light, temperature and water stress [7]. Anthocyanins are proposed to be light attenuators induced in high-light conditions [8], and their biosynthesis is increased by colder temperatures and repressed by higher temperatures via MYB transcription factors [9]. Cold temperatures are known to increase alternative splicing in tubers [10].

Northern latitudes have cooler nights and longer photoperiods with unique UV-B and red to far-red light ratios, all of which influence phenylpropanoid biosynthesis [11,12]. Anthocyanins and flavonols were more abundant in bilberries and white birch from the north of Finland compared to the south [13,14]. Also regulated by environment are carotenoids, C40 isoprene derivatives that function as photoprotectants in plants. Carotenoids increased in kale with increasing temperature, but decreased in spinach [15]. As with phenylpropanoids, light intensity influences carotenoid expression. Larger xanthophyll pools are found in leaves in sun versus shade [16], whereas increased zeaxanthin is associated with cold-hardening in evergreens [17].

Besides their physiological roles, phenylpropanoids and carotenoids influence the nutritional value of potatoes. Phenylpropanoids have multiple health-promoting properties and can function as antioxidants, or have anti-inflammatory, hypotensive, anti-cancer effects or promote cardiovascular health [18-21]. High phenolic potatoes were found to decrease inflammation and oxidative damage in men [22]. Likewise, carotenoids promote cardiovascular health, are chemopreventive, and lutein and zeaxanthin reduce the risk of age-related macular degeneration [23,24].

Most studies on environmental modulation of secondary metabolites have focused on above ground plant parts like leaves, fruits and berries. Less is known about environmental effects on tubers, which are not directly exposed light. Location effects on antioxidants were found in potatoes grown in Texas, and potatoes grown at higher altitudes in Colorado had more phenolics than potatoes grown in Texas [25,26]. Deliberately imposed drought stress was found to increase tuber glycoalkaloid content and have differential effects on tuber metabolites and genes [27,28].

In this study we sought to better understand environmental effects on tuber secondary metabolites, especially in tubers grown under modern agronomic conditions that deliberately minimize stress and maximize yield. Indeed, the role of many secondary metabolites is to allow plants to cope with stress, and tuber metabolites can be manipulated by imposing drought or nutrient stress. However, such treatments result in severe negative effects on yield and are not necessarily informative about secondary metabolism occurring in the more "buffered" conditions a managed crop encounters in contrast to a wild plant under native conditions. Moreover, as underground storage organs, tubers are not directly exposed to light and the soil provides a degree of buffering against various environmental stimuli foliar tissues encounter. Growing tubers in different locations in conventional conditions, allows environment to be used as a tool to study normal fluctuations in tuber secondary metabolites. We suspected such potatoes would show differences in secondary metabolism, but less obvious was the type and magnitude of any such changes and especially what type of relationships would be seen among tuber metabolites. Analyzing gene expression and metabolite pools in potatoes grown in diverse locations could help understand how tuber secondary metabolism is differentially expressed and whether environment affects the nutritional value of a managed tuber crop.

## Results

**Figure 1:**
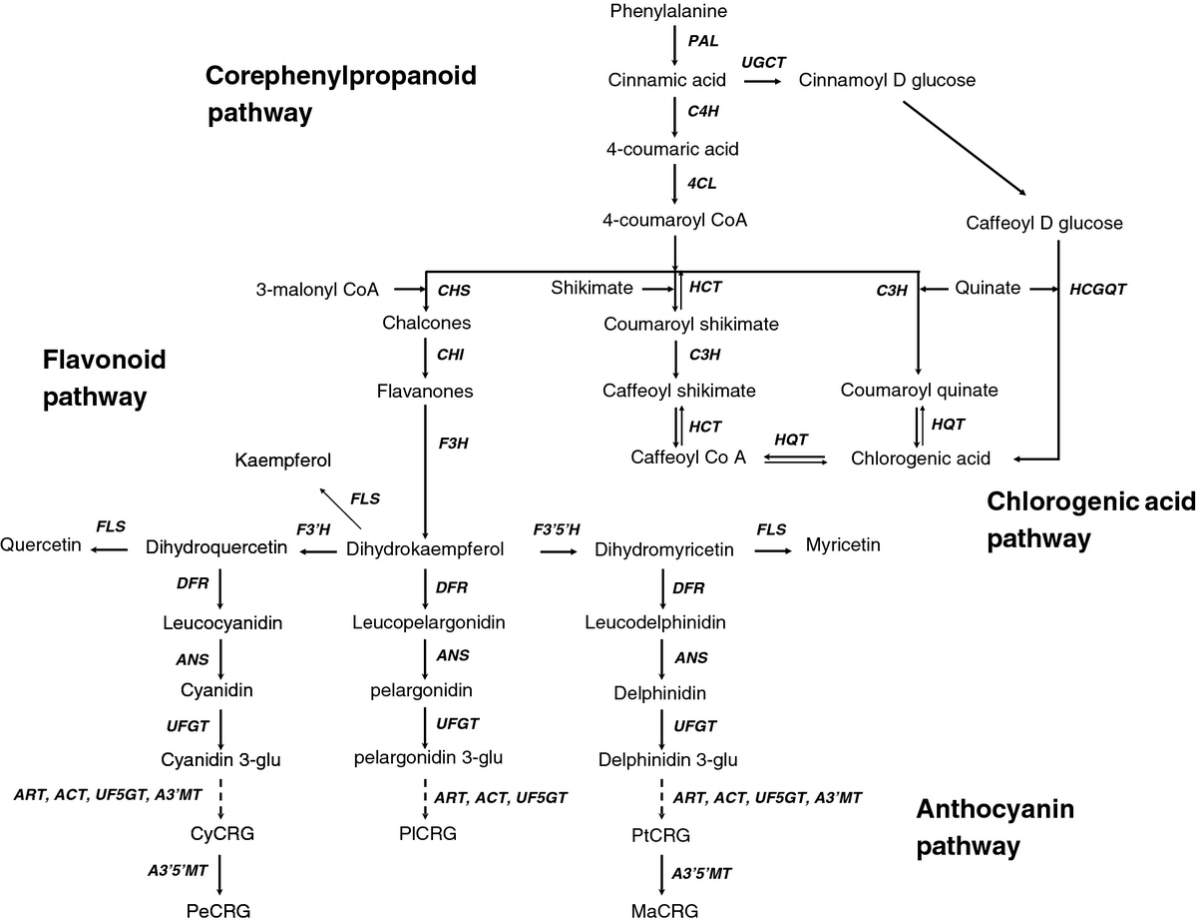
**Phenylpropanoid metabolism**. Abbreviations: PAL, phenylalanine ammonia-lyase; C4H, cinnamate 4-hydroxylase; 4CL, 4-coumaroyl:CoA-ligase; C3H, p-coumarate 3-hydroxylase; HCT, hydroxycinnamoyl Co A shikimate hydroxycinnamoyl transferase; HQT, hydroxyl-cinnamoyl CoA quinatehydroxycinnamoyl transferase; UGCT, UDP glucose:cinnamateglucosyl transferase; HCGQT, hydroxycinnamoylglucose:quinatehydroxycinnamoyl transferase, CHS, chalcone synthase; CHI, chalconeisomerase; F3H, flavanone 3-hydroxylase; F3'H, flavonoid 3' hydroxylase; F3'5'H, flavonoid 3'5' hydroxylase; FLS, flavonol synthase; DFR, dihydroflavonol 4-reductase; ANS, Anthocyanin synthase; UFGT, UDP glucose:flavonolglucosyl transferase; ART, anthocyanin rhamnosyltransferase; ACT, anthocyanin acyltransferase, UF5GT, UDPG flavonoid 5-O-glucosyltransferase; A3'MT, anthocyanin 3'-methyltransferase; A3'5'MT, anthocyanin 3',5'-methyltransferase. CyCRG, PeCRG, PlCRG, PtCRG and MaCRG are coumaryolrutinosideglucosides of cyanidin, peonidin, pelargonidin, petunidin, and malvidin respectively. (Boss, Davies & Robinson, 1996, Gutha et al., 2010, Kroon, Souer, De Graaff, Xue, Mol&Koes, 1994, Niggeweg, Michael & Martin, 2004).

A sizeable majority of total tuber antioxidant capacity is hydrophilic and contributed by phenylpropanoids, whereas carotenoids contribute to tuber lipophilic antioxidant capacity. These compounds are known to be environmentally responsive. Therefore, 'Magic Molly' (MM), a purple-flesh cultivar with especially high amounts of phenylpropanoids [29] was grown in six locations, four in Alaska, and one in Texas and Florida (Table 1). Wiseman, located north of the Arctic Circle, was the northernmost location and Hastings, FL the southernmost. Mature tubers were harvested from each location and phenylpropanoid metabolism (Figure 1) including phenolic acids, hydroxycinnamic acid amides, flavonols and anthocyanins was characterized.

**Table 1:** Latitude and longitude of locations from where the tubers were harvested.

Location	Latitude	Longitude
Wiseman, AK	67.2°	150.1°

Fairbanks, AK	64.5°	147.4°

Palmer, AK	614.3°	149.6°

Juneau, AK	58.2°	134.3°

Lubbock, TX	33.3°	101.5°

Hastings, FL	29.4°	81.3°

Information was collected at http://geonames.usgs.gov/

### Environmental influence on hydroxycinnamic acid metabolism

**Figure 2:**
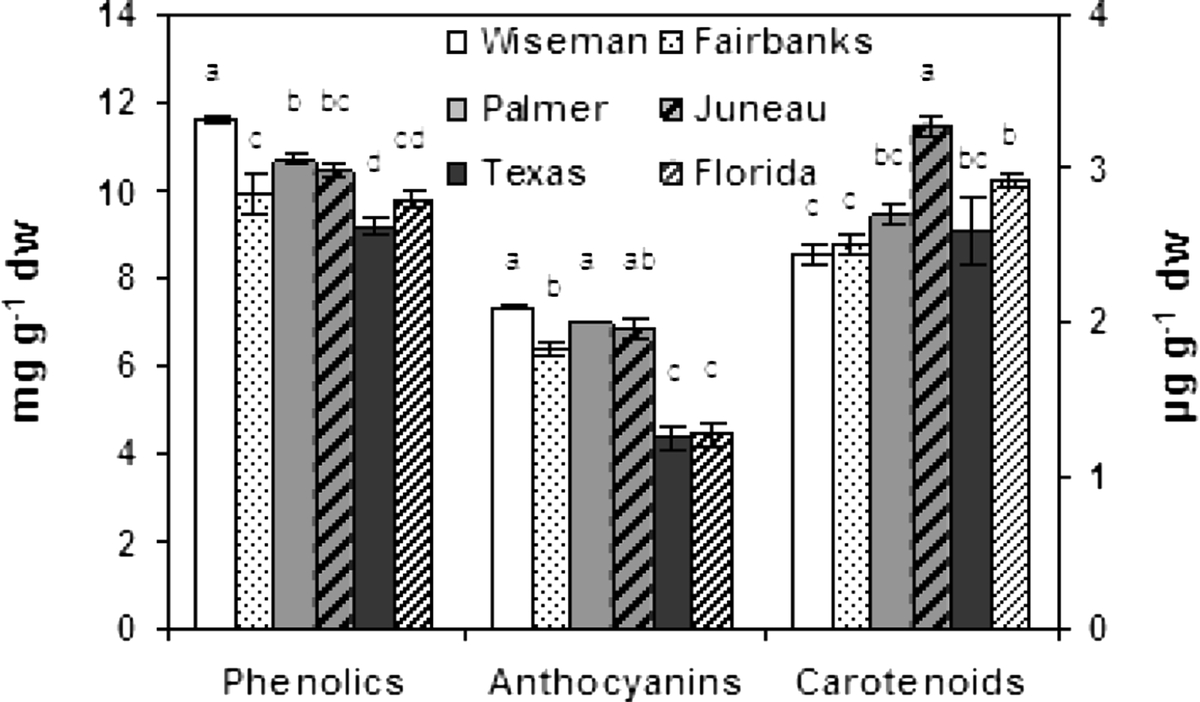
**Totalphenolics and anthocyanins in tubers from six different locations**. The 2^nd ^Y axis shows carotenoid concentrations. Data represents the means ± SE of three biological replicates. Locations with same letter are not significantly different (*p *< 0.05).

**Figure 3:**
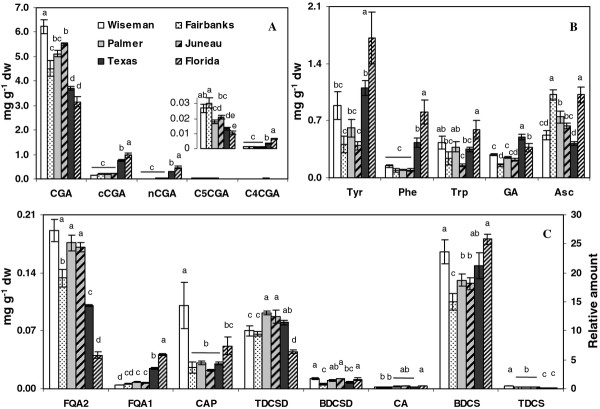
**Phenylpropanoid concentrations in tubers from six locations measured by HPLC**. (**a**) chlorogenic acids with levels of CCGA and C4CGA in the inset. (**b**) aromatic amino acids, glycoalkaloids and ascorbate. (**c**) caffeic acid, feruloyl derivatives, polyamines. CGA, chlorogenic acid; cCGA, crypto chlorogenic acid; nCGA, neochlorogenic acid; C4CGA and C5CGA, chlorogenic acid isoform; FAQ1 and FAQ2, feruloylquinic acid isoforms; CA, caffeic acid; CAP, caffeoylputrescine; TDCSD, tris-dihydrocaffeoyl spermidine; BDCSD, bis-dihydrocaffeoylspermidine; BDCS, bis-dihydrocaffeoylspermine; TDCS, tris-dihydrocaffeoyl spermine; Tyr, tyrosine; Phe, phenylalanine; Trp, tryptophan; GA, glycoalkaloids; Asc, ascorbate. Refer to 2^nd ^Y-axis for CA, BDCS, and TDCS. The data represents the means ± SE of three biological replicates. Locations with same letter are not significantly different (*p *< 0.05)

Overall phenylpropanoid activity in tubers was assessed by measuring total phenolics and total anthocyanins. Results suggested the phenylpropanoid pathway was more active in Alaskan tubers (Figure 2). Total phenolics ranged from 9 to 11.5 mg g^-1 ^DW, a ~28% spread, with tubers from northernmost location (Wiseman) accumulating the most and those from the southernmost locations the least (Figure 2). Changes in individual phenolics were characterized by LCMS. Typically in potatoes, a majority of the soluble phenolics are hydroxycinnamic acid derivatives, and chlorogenic acid (5-O-caffeoylquinic acid; CGA) is the most abundant phenolic. Therefore it was surprising that the CGA variation among locations was considerably greater than the differences in total phenolics, with the Wiseman tubers having an almost two-fold greater amount of CGA than the Florida tubers (Figure 3A). Four additional chlorogenic acid isomers with molecular ions of *m/z *353 (M-H)^- ^were detected and peak assignments (Additional file 1) were based on relative intensity of the MS^2 ^ions and retention time [30]. These four CGA isomers were each less abundant than CGA, with the cis-isomers the least abundant. In contrast to CGA, 3-O and 4-O-CGA were several-fold more abundant in the Texas and Florida samples than in the Wiseman samples. The relative proportions of the chlorogenic acids varied among the locations, with 4-O-CGA only 2% to 5% of CGA amounts in Alaska samples but 20% and 31% of CGA levels in Texas and Florida samples, respectively (Figure 3A). Similarly, 3-O-CGA was less than 0.3% of the CGA amounts in Alaskan samples but 8% and 15% in Texas and Florida samples.

Unlike the chlorogenic acids, no clear trend was observed between southern and northern locations for caffeic acid (Figure 3B). Two additional hydroxycinnamic acid derivatives were identified as feruloylquinic acid (FQA) isomers with molecular ions of *m/z *367 (M-H)^-^(Additional file 1). FQA1 was the least abundant of the two isomers and was present at higher amounts in the Texas and Florida tubers (Figure 3B), whereas the more abundant FQA2 was highest in Alaskan tubers.

Several caffeoyl polyamine derivatives or hydroxycinnamic acid amides (HCAA) were present in tubers. Concentrations of caffeoylputrescine varied over 3-fold among locations but did not obviously relate to latitude (Figure 3B). About a two-fold variation was observed in four dihydrocaffeoyl polyamines among locations, but only tris-dihydrocaffeoyl spermine (TDCS) seemed influenced by latitude.

**Figure 4:**
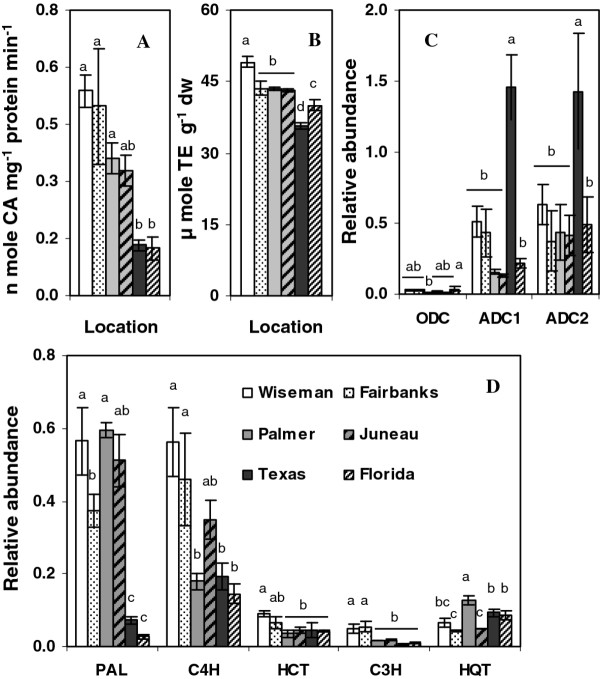
**PAL activity, antioxidant capacity and transcript relative abundance**. (**a**) PAL enzyme activity (**b**) antioxidant capacity in trolox equivalents (**c**) transcript abundance of polyamine genes and (**d**) phenylpropanoid genes measured by qPCR in tubers from six locations. Abbreviations as described in Figure 1. Data represents the means ± SE of three biological replicates. Values with same letter are not significantly different (*p *< 0.05)

Phenylalanine ammonia lyase (PAL) catalyzes the first committed step in phenylpropanoid metabolism with the deamination of phenylalanine to cinnamic acid (Figure 1) and has a key role in regulating carbon flux into the pathway [31,32]. PAL enzyme activity was generally higher in the more northern latitudes (Figure 4A) as were total phenolics (Figure 2). PAL activity in samples from Texas and Florida was 2.5 to 4-fold lower than the samples from Alaska. Interestingly, phenylalanine concentrations were inversely correlated with tuber *PAL*expression and were 4 and 8-fold higher in samples from Texas and Florida than Alaska (Figure 3C). Phenylalanine, along with tryptophan and tyrosine, are aromatic amino acids derived from the shikimate pathway and all three were highest in the Florida tubers (Figure 3C). *PAL *expression was measured by real-time RT-PCR and a relationship was observed between *PAL *transcript abundance, latitude and total phenolics (Figure 4D).

Expression of additional phenylpropanoid genes was measured, and *C4H, HCT *and *C3H *were typically more highly expressed at northern latitudes. The antioxidant capacity of the samples from different locations varied between 35.7 to 49.1 μmole TE g^-1 ^as measured by FRAP assays and was greatest in the Wiseman tubers (Figure 4B). Tubers from the southern locations had lower antioxidant values (35.7 and 39.9 μmole TE g^-1^) than the Alaskan samples, which was consistent with the overall phenylpropanoid activity in the different locations.

**Figure 5:**
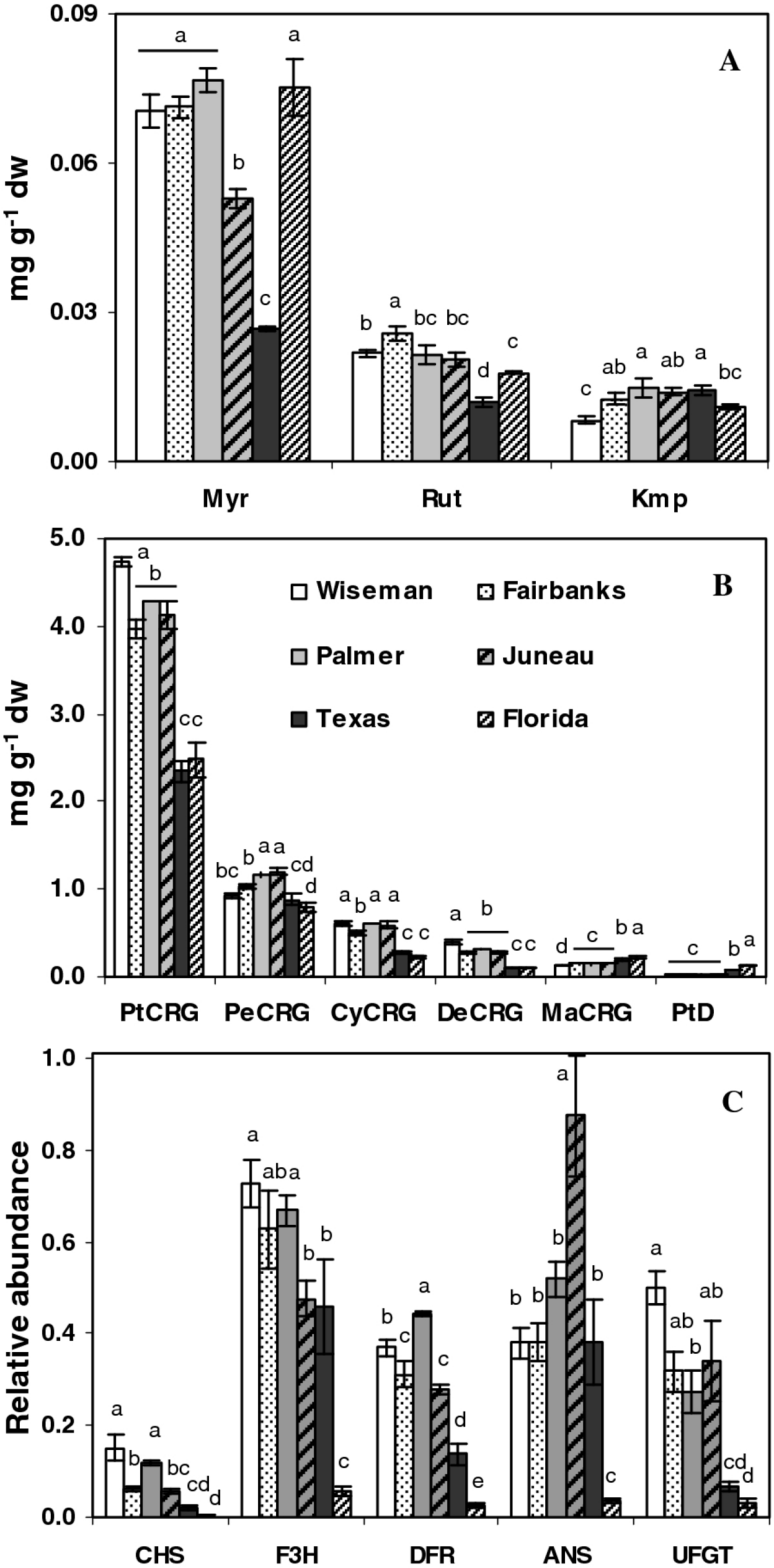
**Correlation analysis of phenylpropanoid transcript and metabolite levels in tubers**. Pearson correlation coefficients were calculated and used to generate a heatmap in which positive correlations are shown in red and negative correlations in blue. Most abbreviations are listed in Figure 1, Figure 3, Figure 6 and supplementary Table **1**. PALe is PAL enzyme activity. Phn, total phenolics; Ant, total anthocyanins; Crt, total carotenoids; FRAP, antioxidant capacity; Glu, glucose, Suc, sucrose, GA, glycoalkaloids, Asc, ascorbate.

Few of the genes involved in potato HCAA metabolism have been identified, so expression of genes that mediate entry into the putrescine pathway were measured. Putrescine can be synthesized from arginine via arginine decarboxylase (ADC) or ornithine by ornithine decarboxylase (ODC) [33]. Primers were designed against two potato *ADC *genes found in database searches, along with ODC. Both *ADC *genes were more highly expressed than *ODC*, but the expression of these genes did not correlate with the amounts of caffeoyl polyamines, suggesting any transcriptional control would have to occur downstream in the pathway (Figure 4C and Figure 5).

There was no apparent relationship between ascorbic acid and latitude, and about a two-fold difference was seen among the locations with the Florida and Fairbanks samples having the most (Figure 3C). Glycoalkaloids (solanine and chaconine) ranged from 0.16 to 0.5 mg g^-1 ^and were lowest in Alaskan samples. Texas and Florida samples accumulated 120% and 60% higher amounts of glycoalkaloids.

### Flavonoids

**Figure 6:**
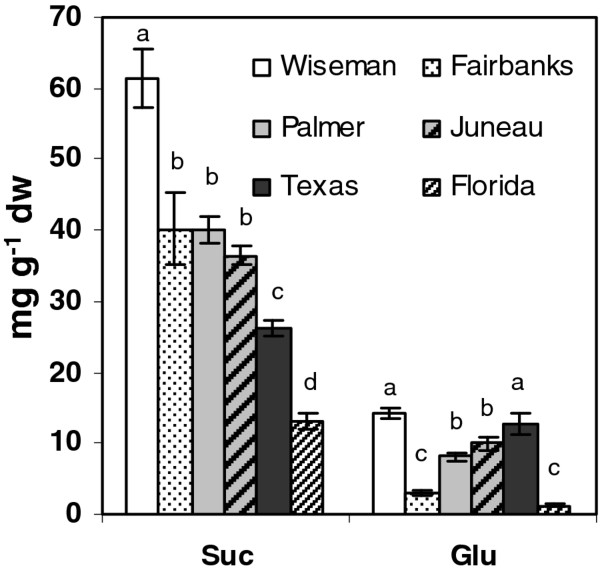
**Flavonoid concentrations and gene expression**. (**a**) flavonol (**b**) anthocyanin concentrations and (**c**) flavonoid gene expression in tubers from six locations. PtCRG, PeCRG, CyCRG, DeCRG, MaCRG are 3-coumuryol rutinoside 5-glucoside conjugates of petunidin, peonidin, cyanidin, delphinidin, and malvidin respectively; Ptd, petunidin derivative; Myr, myricetin-3-*O*-rutinoside; Rut, quercetin-3-*O*-rutinoside; Kmp, kaempferol-3-*O*-rutinoside. The data represents the means ± SE of three biological replicates. Locations with same letter are not significantly different (*p *< 0.05)

Environmental effects on tuber flavonoids, which are further downstream in the phenylpropanoid pathway (Figure 1), were examined. Tubers accumulated low quantities of flavonols. Three flavonols were quantified, myricetin-3-*O*-rutinoside (Myr), quercetin-3-*O*-rutinoside (Rut) and kaempferol-3-*O*-rutinoside (Kmp), of which Myr was the most abundant (Figure 6A). Flavonols varied over two-fold among locations but did not appear associated with latitude. Flavonol concentrations were somewhat comparable among most locations, but substantial differences were seen in some locations, such as the ~3-fold lower amounts of Myr in the Texas samples (Figure 6A). Rutin was also lower in Texas samples (0.012 mg g^-1^) compared to other locations (0.017 to 0.025 mg g^-1^). The total flavonol levels were only 50% in samples from Texas compared to other locations including Florida.

In contrast to the flavonol branch, the anthocyanin branch of the flavonoid pathway was very active. Total anthocyanins in the Wiseman tubers were ~66% more abundant than in those from the South. Total anthocyanins reached ~6.3-7.3 mg g^-1 ^in samples from the four Alaskan locations and 4.4 mg g^-1 ^in samples from Texas and Florida (Figure 2). Over ten individual anthocyanins were identified by LCMS based on retention time, precursor ion, product ion and neutral loss analyses. The most abundant are listed in Additional file 1. Petunidin-3-coum-rutinoside-5 glucoside (PtCRG) was the major anthocyanin (53% to 64% of total anthocyanins) followed by peonidin-3-coum-rutinoside-5 glucoside (13% to 20%; PeCRG; Figure 6B). Most of the major anthocyanins such as PtCRG, cyanidin-3-coum-rutinoside-5-glu, and delphinidin-3-coum-rutinoside-5-glc were significantly lower in samples from Texas and Florida compared to Alaska. However, two less abundant anthocyanins, malvidin-3-coum-rutinoside-5 glucoside and a petunidin derivative with a molecular ion of *m/z *933 (M + H)^+ ^showed an opposite trend (Figure 6B).

### Flavonoid gene expression

Chalcone synthase (CHS) catalyzes the first committed step (Figure 1) in the synthesis of flavonoids, compounds that share a 15 carbon skeleton. *CHS *transcript was more strongly expressed in the samples from northern latitudes (Figure 6C) and correlated with tuber anthocyanin content (r = 0.88) and *PAL *expression (r = 0.88; Figure 4D and Figure 5). Flavanone 3-hydroxylase (F3H) is also necessary for production of both flavonols and anthocyanins and catalyzes formation of dihydroflavonols from flavanones. *F3H *transcript abundance generally correlated with CHS and flavonoids (Figure 5), except for the Texas samples which had similar amounts of phenolics and anthocyanins to the Florida samples (Figure 2), yet several-fold higher expression of *F3H*. DFR (dihydroflavonol 4-reductase) catalyzes the first step in the branch to the colorless leucoanthocyanidins by the reduction of dihydroflavanols. *DFR *expression was higher in the northern samples and correlated with *PAL, CHS *and anthocyanin levels (Figure 6C and Figure 5). The dioxygenase, anthocyanidin synthase (ANS), converts colorless leucoanthocyanidins to anthocyanidins. *ANS *expression was higher in the northern than the Florida samples, but the relative expression in the Juneau and Texas samples poorly correlated with anthocyanin concentrations. The final late stage anthocyanin gene examined was UDP glucose:flavonolglucosyl transferase (*UFGT*) which strongly correlated with anthocyanin levels (Figure 5) and was several-fold less expressed in the Texas and Florida samples that had the lower anthocyanin levels (Figure 6C). Overall, flavonoid gene expression was much lower in the Florida samples.

### Transcript and metabolite correlation analysis

Correlation matrices were used to compare gene expression and metabolite abundance. In addition to individual metabolites, total anthocyanins, total phenolics and total carotenoids were also included, along with PAL enzyme activity (PALe) and FRAP antioxidant capacity. When Pearson correlation coefficients were expressed as a heatmap, it was clear that a majority of the factors had either positive or negative correlations (see r values in Figure 5). Phenylpropanoid gene expression generally correlated well with phenylpropanoid concentrations, which in this genotype were largely chlorogenic acids or anthocyanins. The most abundant chlorogenic acid, CGA, was positively correlated with phenylpropanoid pathway genes, but the less abundant 3- and 4CGAs were negatively correlated with both CGA and overall phenylpropanoid transcript and metabolite abundance. A similar trend was seen for two ferulic acid isomers. Likewise the most abundant anthocyanins were positively correlated with phenylpropanoid transcript abundance, but those present in substantially lower amounts showed negative correlations. UFGT is responsible for the glycosylation of anthocyanidins and is active late in the phenylpropanoid pathway. CGA is synthesized earlier in the pathway and is not in the anthocyanin branch, but CGA concentrations strongly correlated with UFGT expression. This suggests that if downstream branches of the phenylpropanoid pathway are more active it will also influence other branches.

### HQT expression

Curiously, of the ten phenylpropanoid genes analyzed, *CGA *concentrations showed the weakest correlation with *HQT*, the putative terminal enzyme in the CGA pathway, but strongly correlated with the other phenylpropanoid genes (Figure 5). CGA is thought to be synthesized by HQT, although additional pathways may contribute to its synthesis [34,35].

### Sugar analysis

**Figure 7:**
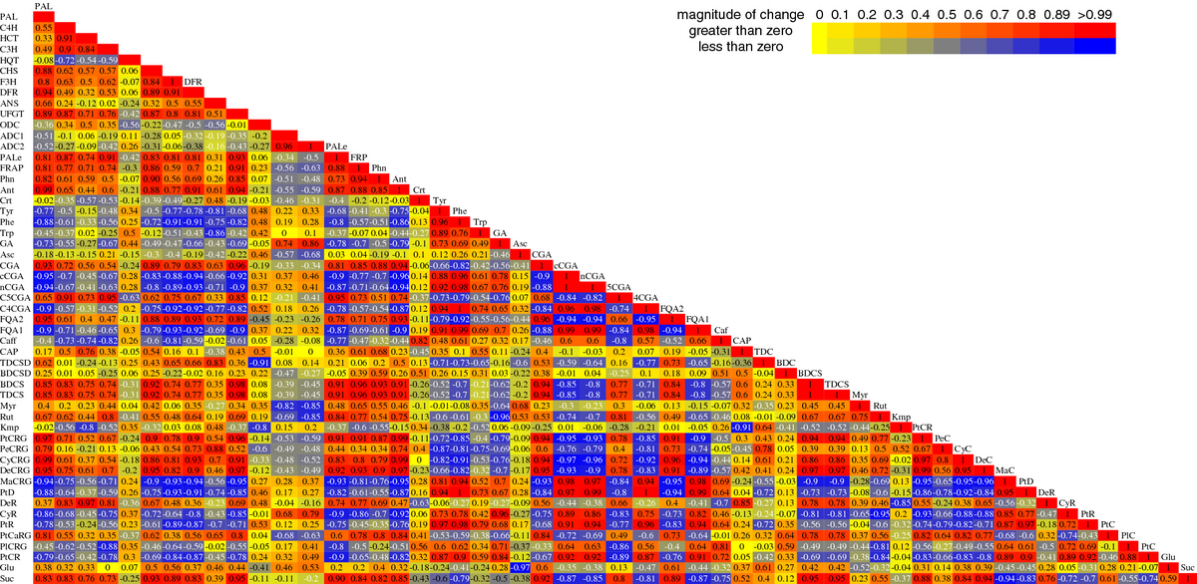
**Levels of sucrose (Suc) and glucose (Glu) in tubers from six locations**. The data represents the means ± SE of three biological replicates. Locations with same letter are not significantly different (*p *< 0.05)

Sugars such as sucrose and glucose are known to induce anthocyanins and chlorogenic acid in plants by activation of structural genes [36,37]. Therefore, one potential mechanism by which environment may modulate tuber phenylpropanoid expression could be through sugars. Expression of five genes involved in primary metabolism were examined, one each in the shikimate and glycolytic pathway and three starch metabolism genes (Additional file 2). Only sucrose synthase showed a clear trend to higher expression in the more northern latitudes. Sucrose and glucose concentrations were measured in samples from different locations and sucrose levels were higher than glucose in all samples, ranging from 13-60 mg gm^-1 ^(Figure 7). Sucrose levels showed a clear decreasing trend from north to south locations and were significantly higher in samples from northern Alaska and lowest in the Florida samples. Glucose levels ranged from 1.2-14 mg gm^-1^, and were also highest in Wiseman and lowest in Florida samples. Tuber sucrose positively correlated (Figure 5) with total phenolics and anthocyanins and the lower expression of the anthocyanin genes in Florida samples may be at least partly explained by the lower sucrose content.

### Promoter analysis

The coordinated expression of tuber phenylpropanoid genes suggested their promoters may share common elements, including some that are environmentally responsive. Promoter analysis was conducted on a ~1500 bp region upstream of the ATG translation start codon to identify environmentally responsive cis-elements in nine of the studied phenylpropanoid genes (Table 2). Promoter sequence was not available for *UFGT*. Numerous shared cis-acting regulatory elements were identified, and the most abundant motifs were light-responsive elements such as Box 4, G box, GT1, L box, and I box (Table 2). Box 4 and I box were present in all promoters. A G box was observed in all promoters except *C4H *and *C3H*, and was abundant in *HCT, CHS *and especially *DFR*. A GT1 motif was also observed in all promoters except *C4H *and *HCT*. A circadian element was present in *PAL, C4H, HCT *and *C3H, CHS *and *ANS*. TC rich repeats, a defense and stress response element, was one of the most represented elements in all the genes except *CHS*. Interestingly, the low temperature response element (LTR) was only present in *HQT*. A heat stress responsive element was present in the all the promoters except *HQT *and *CHS *(Table 2). A methyl jasmonate responsive element, CGTCA motif, was present in *PAL, HCT, CHS *and *DFR*. Overall *DFR *had the most occurrences of the cis-elements listed in Table 2 with a total of 26. The two most represented cis-elements were the TC-rich repeats (24) and G-Box (23). All cis-elements were present in more than one gene, except for EIRE and LTR which were present only in *HQT*.

**Table 2:** List of putative regulatory elements present in promoters of nine genes.

Motif	Sequence	Function	PAL	C4H	HCT	C3H	HQT	CHS	F3H	DFR	ANS
Box 4	ATTAAT	light responsiveness	3	4	2	1	2	1	4	1	2

Box I	TTTCAAA	light responsive element	1	3	2	2	1	1	3	2	2

CGTCA-motif	CGTCA	MeJA-responsiveness	2	0	1	0	0	2	0	3	0

EIRE	TTCGACC	elicitor-responsive element	0	0	0	0	1	0	0	0	0

ERE	ATTTCAAA	ethylene-responsive element	1	2	2	1	0	0	1	0	0

G-Box	CACGTN	light responsiveness	2	0	4	0	1	5	2	8	1

GA-motif	AAGGAAGA	part of a light responsive element	0	0	0	0	2	0	1	0	1

GATA-motif	GATAGGA	part of a light responsive element	0	2	0	0	0	1	3	1	0

GT1-motif	GGTTAA	light responsive element	1	2	2	1	2	1	1	2	2

HSE	AAAAAATTTC	heat stress responsiveness	1	2	2	1	0	0	1	3	3

I-box	AGATAAGG	part of a light responsive element	1	2	0	0	1	0	3	2	2

L-box	TCTCACCAACC	part of a light responsive element	1	1	1	1	0	0	0	1	0

LTR	CCGAAA	low-temperature responsiveness	0	0	0	0	1	0	0	0	0

TC-rich repeats	RTTTTCTTMM	defense & stress responsiveness	5	1	5	2	3	0	4	3	1

TCT-motif	TCTTAC	part of a light responsive element	1	2	3	1	0	1	0	0	0

Circadian	CAANNNNATC	circadian control	1	2	1	3	0	1	0	0	3

Number of times the element present in a specific promoter is given in the gene column. Standard IUPAC nucleotide codes were used for variable bases

### Carotenoids

**Figure 8:**
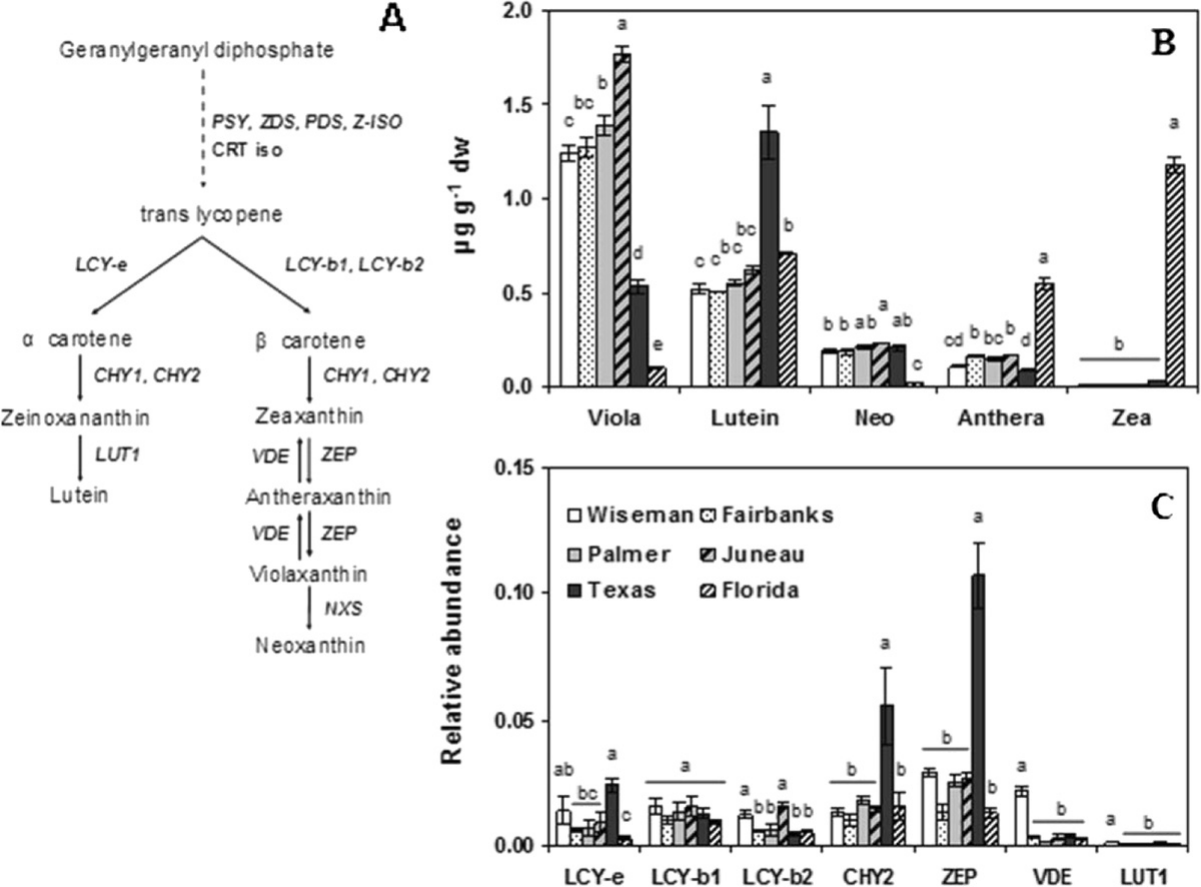
**Tuber carotenoid concentrations and gene expression**. (**a**) Carotenoid biosynthetic pathway in plants. PSY1 and 2, phytoene synthase isoforms; PDS, phytoenedesaturase; Z-ISO, ζ -carotene isomerase; ZDS, ζ -carotene desaturase; CRT iso, carotene isomerase; LCY-b1 and b2, lycopene β cyclase isoforms; LCY-e, lycopene ε cyclase; CHY1, and 2, β carotene hydroxylase isoforms, ZEP, zeaxanthin epoxidase; VDE, violaxanthin de-epoxidase; NXS, neoxanthin synthase, LUT1, carotene hydroxylase (Diretto et al., 2006, Zhu et al., 2010). (**b**) concentrations of five anthocyanins measured by HPLC in tubers from six locations. (**c**) Expression of seven genes downstream of lycopene. Viola, violaxanthin; Neo, neoxanthin; Anthera, antheraxanthin; Zea, zeaxanthin. The data represents the means ± SE of three biological replicates. Locations with same letter are not significantly different (*p *< 0.05)

While these hydrophilic phenylpropanoid derivatives are by far the largest contributors to tuber antioxidant capacity, lipophilic antioxidants are also present. Modest differences in total carotenoid concentrations occurred among locations, ranging from 2.45 to 3.3 μg gm^-1 ^and differences did not seem related to latitude (Figure 2). Despite the modest variation in total carotenoids, LCMS profiling of the carotenoids revealed striking differences (Additional file 3). Violaxanthin was the major carotenoid in all Alaskan samples (1.2-1.8 μg gm^-1^), contributing more than 50% of the total carotenoids in these tubers (Figure 8B). However, Florida tubers contained only 0.1 μg gm^-1 ^violaxanthin, which comprised only 3.5% of the total carotenoids, whereas it contributed 20% in the Texas potatoes. Lutein dominated in Texas tubers and was present at a two-fold higher concentration than violaxanthin and contributed 50% of total carotenoids (Figure 8B), but in other locations lutein contributed 19% to 25%. While the Alaskan tubers contained at least two-fold greater amounts of violaxanthin than lutein, the Florida samples had seven-fold greater amounts of lutein than violaxanthin. Florida tubers also contained considerably less neoxanthin and more antheraxanthin than the other samples. Finally, the Florida tubers contained much higher amounts of zeaxanthin, which contributed about 40% of the total, whereas it was less than 1% in samples from all other locations.

### Carotenoid gene expression

Because of the surprising differences in carotenoid content in the same genotype, carotenoid gene expression was examined. Florida potatoes had higher concentrations of zeaxanthin and antheraxanthin than the downstream products violaxanthin and neoxanthin (Figure 8A and 8B). This difference might be explained by lower zeaxanthin epoxidase (ZEP) activity, however while Florida tubers did have lower expression of *ZEP *than the other samples the difference was not statistically significant. Moreover, *ZEP *was most highly expressed in Texas tubers, which however had lower amounts of violaxanthin and average neoxanthin. Lycopene ε cyclase (*LCY-e*) catalyzes entry into the α-carotene branch of the carotenoid pathway and was highest in the Texas tubers, which had the most lutein. However, carotene hydroxylase (*LUT1*) and lycopene β cyclase isoform (*LCY-b1 *and *LCY-b2*) expression, which is downstream of *LCY-e *in the lutein pathway, were not higher in the Texas material. A good correlation between carotenoid gene expression and carotenoid pools was not generally observed (Figure 8 and Additional file 4), and transcriptional differences did not appear to account for the differing carotenoid profiles.

## Discussion

We conducted a targeted analysis of tuber secondary metabolism in potatoes grown in a modern agricultural system that maximizes yield and essentially ameliorates the extreme environmental stresses a wild plant would naturally encounter. To better understand the metabolism of stress inducible compounds when stresses are deliberately minimized, secondary metabolites were evaluated in potatoes grown in multiple locations that encompass the general range of environmental conditions a U.S. crop would encounter. Environmental variables among locations included marked differences in day and night temperatures, light intensity, day length, cloud cover, humidity, soil type, time of planting and coastal versus inland locations. The secondary metabolites assessed are of particular interest because, in addition to their physiological roles, they are major determinants of the nutritional value of potatoes. Results showed that environmental influences occurring under managed conditions are sufficient to significantly impact the nutritional value of potatoes, with a two-fold or greater change seen for some phytonutrients. Importantly, over two-fold differences were seen in some metabolites between the Texas and Florida locations, showing significant environmental variation can occur in typical growing locations. Such substantial changes would be predicted to be especially relevant for staple crops because the high consumption means even modest alteration in phytonutrients will have a dietary impact.

We suspected the Alaskan potatoes would have higher amounts of some secondary metabolites, given the effects light and temperatures are known to have on plant phenylpropanoid and carotenoid pathways. The Wiseman location is north of the Arctic Circle and does not experience darkness in mid-summer. Total phenolics were highest in Wiseman and generally higher in the Alaskan samples than the southern samples. The spread from low to high was a somewhat surprisingly modest 28% as measured by the FC method. However, when individual phenylpropanoids were measured by HPLC substantial differences were found, including a two-fold difference in CGA. This is significant because CGA is typically most abundant phytonutrient in tubers and has been reported to constitute about 90% of tuber phenolic content in some genotypes [38]. Stimuli known to increase CGA biosynthesis in potatoes include light [4], while seasonal variation in CGA content occurs in evergreens [39]. Interestingly, the lower CGA content of the southernmost samples was partly offset by the several-fold higher amounts of 3CGA and 4CGA (Figure 3A).

*HQT *expression did not correlate with CGA concentrations or other phenylpropanoid pools (Figure 3A, 4D and Figure 5). Curiously, a better correlation was observed between all of the other phenylpropanoid genes and CGA, than between *HQT *and CGA, as calculated using Pearson product moment correlation coefficients (Figure 5). Gene expression analysis was conducted on mature tuber tissue sampled at the end of the season and therefore would not detect transient changes in expression occurring earlier in development, prior to the sampling point. However, these analyses do allow comparison of gene expression and metabolite levels in mature tubers at the same time point. CGA is synthesized by *HQT*, although additional pathways are possible [34,35]. Promoter analysis identified two elements (EIRE and LTR) that were present in *HQT*, but not the other phenylpropanoid genes. Whether these contribute to the difference in *HQT *expression relative to the other phenylpropanoid genes is unclear. It has not yet been definitively shown that *HQT *is the only or even major route to CGA synthesis in tubers, although based on results with tomato this seems probable [35]. A weak correlation between *HQT *mRNA expression and CGA concentrations was previously reported in coffee grains, artichoke leaves and drought stressed potatoes [27,40,41].

The strongest correlation between core phenylpropanoid pathway gene expression and metabolite pools occurred with *PAL*, emphasizing the importance of this gene in modulating tuber phenylpropanoid metabolism (Figure 5). The lower phenylalanine pools in samples with higher PAL gene and enzyme activity is consistent with more flux through the pathway. Similarly, reduced PAL activity and higher levels of phenylalanine in the southern samples may reflect less carbon flow into the phenylpropanoid pathway and slower turnover of phenylalanine. The strong correlation between *UFGT *expression and the more abundant tuber hydroxycinnamic acids like CGA and FQA2 suggests that increased anthocyanin biosynthesis may lead to increased amounts of upstream phenylpropanoids outside of the anthocyanin branch. This could reflect coordinate up-regulation of the overall phenylpropanoid pathway in response to increased anthocyanin biosynthesis. The anthocyanins would act as a carbon sink and increase carbon flow from the shikimate pathway into the phenylpropanoid pathway. Such a mechanism could explain why anthocyanin-rich potatoes also tend to have greater amounts of colorless phenolics like CGA [29]. An interesting finding was the inverse relationship seen between some isomers or among closely related compounds in which the more abundant isomers or family member were positively correlated with phenylpropanoid transcript abundance, but the less abundant ones were negatively correlated. This was observed among the chlorogenic acids, ferulic acids and anthocyanins and shows that tubers have the ability to increase or decrease individual phenylpropanoids even though the pathway is coordinately regulated. This may also allow tubers to partially, but not completely, compensate for a decrease in certain compounds. For example, the Alaskan samples had higher amounts of CGA, but the southern tubers higher amounts of 3- and 4CGA (Figure 3A) and CGA pools inversely correlated with nCGA and cCGA, which were strongly correlated with each other.

The most abundant anthocyanins strongly correlated with *PAL *expression and with each other (Figure 5) and were significantly higher in Alaskan samples (Figure 2 and Figure 6B) with over a two-fold difference seen in petunidin-3-coum-rut-5-glu (PtCRG), the most abundant anthocyanin. However, expression of individual anthocyanins did not necessarily parallel total anthocyanin levels. The Wiseman samples had the most PtCRG and total anthocyanins, but samples from the Southern U.S. had significantly higher amounts of two less abundant anthocyanins, PtD and MaCRG (Figure 6B). This suggests that individual anthocyanins are differentially synthesized in response to environmental signals. These results are consistent with a report of higher anthocyanins in potatoes grown in Colorado than Texas [25]. Anthocyanin content was also found to differ significantly in Bog Bilberries grown in north or south Finland [13].

Anthocyanins are regulated at transcriptional and translational levels, and are influenced by light, temperature and biotic stresses [2,42-44], and accumulation is favored under lower temperatures [45]. Anthocyanins can decrease under higher temperatures [42] and the mechanism involves both anthocyanin degradation and a decrease in transcription [46]. Consistent with transcriptional control of tuber anthocyanins, samples from Texas and Florida had lower expression of the key flavonoid genes *CHS, DFR *and *UFGT *that correlated with anthocyanin content (Figure 6C and Figure 5).

Sucrose, but not glucose concentrations, positively correlated with the most abundant phenolic acids and anthocyanins and with phenylpropanoid gene expression except for *HQT *and *ANS*, but did not correlate with carotenoids or early polyamine pathway genes. Sucrose had an inverse correlation with the shikimate derived amino acids (Figure 5), which is consistent with some previous reports [47,48]. These amino acids were positively correlated with each other, suggesting coordinate regulation.

A surprising finding was how carotenoid expression varied among locations. Carotenoid composition rather than the total carotenoid content showed the greatest differences (Figure 2 and Figure 8B). The carotenoid profiles seen in HPLC chromatograms varied enough to appear to be from different genotypes, with only trace amounts of some compounds present in one location but abundant in another (Additional file 3). For instance, in the Alaskan samples violaxanthin was the predominant carotenoid, but ten-fold less was present in Florida tubers, which instead had higher amounts of zeaxanthin, which in turn was only present in trace amounts in all the other locations (Figure 8B). This might be due to the xanthophyll cycle in which zeaxanthin and violaxanthin are intercoverted to protect photosystem II from excessive light energy and higher temperatures [49]. The Florida potatoes also had higher amounts of antheraxanthin, an intermediate in the violaxanthin/zeaxanthin reversible interconversion. High light intensity or temperature leads to increased formation of zeaxanthin by violaxanthin de-epoxidase (VDE), whereas low light stimulates the violaxanthin formation by zeaxanthin epoxidase [50]. However, neither *ZEP *nor *VDE *was more highly expressed in the Florida potatoes than other samples, suggesting the differences were not due to transcriptional control of these two genes. Ascorbate has been shown to stimulate the de-epoxidation of violaxanthin to zeaxanthin [51,52] and the Florida samples had greater amounts of ascorbate (Figure 3D). This might favor zeaxanthin formation, but Fairbanks tubers had similar amounts of ascorbate yet only trace amounts of zeaxanthin.

The Texas potatoes were unique in that they had a more than double the amount of lutein, indicating that the α-carotene branch of the pathway was more active. A branch point in the carotenoid pathway occurs at lycopene, which is the substrate for two competing enzymes, LYC-e and LYC-b (Figure 8A). Among the carotenoid genes examined, *CHY2, LCYe *and *ZEP *showed strong correlations (Additional file 4). LCY-e transcript was more highly expressed in the Texas potatoes and catalyzes the ε cyclization of lycopene. The *LUT1 *product catalyzes the terminal step in the pathway, but *LUT1 *was not more highly expressed in the high lutein tubers. A poor correlation between *LUT1 *expression and lutein content was seen in potatoes silenced for *LCY-e *[53] and strong correlations have generally not been reported between transcript levels of genes downstream of lycopene and individual carotenoids [54,55]. The Texas potatoes did not have higher concentrations of any carotenoid other than lutein, yet they showed the highest expression of *ZEP *and *CHY2 *in addition to *LCY-e*. A recent study suggested increased carotenoids in potatoes is due to increased metabolic flux and found correlations between carotenoid genes and metabolites [56], which suggests transcript abundance does influence carotenoid concentrations among cultivars and differs from our study that examined expression within the same genotype in different conditions.

An interesting question is to what extent the tuber directly perceives environmental cues that alter carotenoid or phenylpropanoid composition versus the foliage modulating tuber expression. Several phenylpropanoid gene promoters are known to have light regulatory elements such as Box 4, G box, I box, and L box [57-59]. These elements were also present in most potato phenylpropanoid gene promoters but their role in tubers not exposed to light is unclear.

The observed changes in potato phytonutrients show a potential to customize the nutritional value of a potato crop by choice of cultivar and environment, which may become increasingly relevant with advances in nutrigenomics. For example, the markedly higher anthocyanin amounts in the Alaskan potatoes provide a rationale to take advantage of local environmental conditions by growing red- or purple-flesh potatoes. White-flesh potatoes grown in Alaska would not provide significant anthocyanins, which also illustrates that there are genotypical components to the response to environmental signals. Similarly, the higher amounts of lutein in the Texas potatoes and zeaxanthin in the Florida potatoes raise questions about the extent to which growth can be manipulated to give a desired phytonutrient profile.

## Conclusions

This study showed that even under conditions that minimize environmental stress, significant effects on secondary metabolites occurred and tuber secondary metabolism is quite plastic. This plasticity under non-extreme conditions suggests that a more complete understanding of tuber secondary metabolism regulation may allow phytonutrient content to be optimized during growth, while protecting yield. Despite the growing body of literature on environmental influences on phytonutrient content, we are aware of no staple crop that is deliberately managed to maximize phytonutrients. By profiling a broad swath of tuber phenylpropanoid metabolism it was possible to observe how changes in one compound or compound class affected the overall pathway. Interesting relationships were identified, such as the inverse relationship among many closely related compounds and between phenylalanine and anthocyanin pools. A positive correlation was observed between sucrose and anthocyanins and phenolics, but not with carotenoids (Figure 5), suggesting sucrose may be one regulatory factor for tuber phenylpropanoid synthesis. Tuber phenylpropanoid metabolism responded in a coordinated manner to environmental factors, but a more precise understanding is needed of the steps between signal perception and induction of phenylpropanoid genes.

## Methods

### Plant material

The cultivar 'Magic Molly' is a seedling selection by William Campbell from an open pollinated fruit of Red Beauty at the Plant Materials Center, Palmer Alaska. Seed source can have a large effect on plant performance. To eliminate any variation resulting from seed, all tubers were obtained from Cornell. Seed was planted at four different locations in Alaska, Lubbock, plus Dalhart, Texas in 2007 and then in Hastings, Florida 2008 to obtain additional data from a Southern location (Table 1). Local best management practices were used at each location and the crop was grown by local experts. Climate and soils varied widely across locations. Tubers were planted on the standard date for each location, which ranged from mid-winter in Florida until late spring in Alaska. In-row spacing ranged from 9-11 inches and row spacing from 28-30 inches. Mature tubers were harvested at the end of the growing season, and longitudinal slices were taken from the top, bottom and middle of each tuber, pooled, quickly frozen in liquid N and freeze-dried. Slices were ca. 2 mm in thickness and included periderm only around the periphery of the slice. Three independent biological replicates were used per location. The dried samples were homogenized in a coffee grinder and the powder stored at -80°C until used.

### Phenolic extraction and quantification

Phenolics were extracted as described [60], except fifty mg of freeze dried powder was extracted with a total 1.5 ml of 50% methanol containing 2.5% meta phosphoric acid (Sigma, St. Louis, MO), and 1 mM EDTA in a BeadBeater (Biospec, Bartelsville, OK). Total phenolics were quantified using Folin-Ciocalteau (FC) reagent [61]. Absorbance was read at 755 nm and phenolics quantified as gallic acid equivalents. For LCMS analysis, the extract was concentrated in a Speed Vac (Thermo Savant, Waltham, MA) prior to injection. An Agilent 1100 HPLC system equipped with a quaternary pump, refrigerated autosampler, and column heater was used with DAD and SL ion trap using an ESI source in both positive and negative ion mode. A 100 × 4.6 mm, Onyx monolithic C-18 (Phenomenex) was used at 35°C with a flow rate of 1 ml min^-1 ^and a gradient elution of 0-1 min 100% A, 1-9 min 0-30% B, 9-10.5 min 30% B, 10.5-14 min 35-65% B, 14-16 min at 65-100% B, 16-16.5 min 100% B (Buffer A: 10 mM formic acid pH 3.5 with NH_4_OH; Buffer B: 100% methanol with 5 mM ammonium formate. Phenolic levels were estimated based on a six-point calibration curve developed using commercial standards and gallic acid as an internal standard. CGA and its isoforms were quantified at 320 nm as CGA equivalents, feruloylquinic acid isoforms at 320 nm as ferulic acid equivalents, ascorbic acid at 244 nm, phenylalanine at 210 nm and tyrosine and tryptophan at 280 nm. Caffeoyl putrescence and dihydrocaffeoyl polyamines were quantitated at 210 nm as dihydrocaffeic acid equivalents where the polyamine part was not considered or expressed as normalized, relative mass spec units if a pure peak was not available for UV analysis [60]. Flavonols were quantified at 360 nm as rutin equivalents, and glycoalkaloids at 210 nm as chaconine equivalents. If standards were commercially available, identification was based on retention time and MS data, otherwise tentative assignments were made based on UV and MS data. Retention times and MS data for compounds analyzed are shown in Additional file 1. MS^2 ^ions are listed in order of abundance and relative amounts are shown only if ions were used to discriminate among possible isomers [62].

### Anthocyanin extraction and quantification

For anthocyanin analysis, 50 mg of freeze-dried sample was extracted three times with a total 2 ml of 50% methanol, and 2.5% formic acid with 10 μg of ferulic acid added as an internal standard. Anthocyanins were analyzed on Agilent 1100 LCMS system with the ion trap ESI source in positive mode and a 100 × 3 mm, Onyx monolithic C-18 (Phenomenex) column maintained at 35°C and a flow rate of 0.75 ml min^-1^. Elution was with a gradient of 3-20% B at 0-15 min, 20-35% B from 15-20 min (Buffer A: 10% formic acid, 0.05% TFA; Buffer B: Acetonitrile with 10% formic acid and 0.05% TFA). Anthocyanins were identified using mass data, and quantified at 525 nm as pelargonidin equivalents.

### Carotenoid extraction and quantification

100 mg of powder as extracted with 300 μl methanol containing 200 ng of β-apo-caroten-8-al as internal standard for each sample and 300 μlTris-HCl (50 mM, pH 7.5, 1 M NaCl). Carotenoids were partitioned by adding 800 μl chloroform and vortexing for 10 min, centrifuged 5 min at 4°C and the aqueous phase reextracted with 800 μl chloroform. The combined chloroform extracts were dried in a Speed Vac, then resuspended in 70 μl methanol-ethyl acetate (4:1) containing 0.1% butylated hydroxyl toluene and 30 μl injected onto a 4.6 × 250 mm YMC C30 column (Milford, Massachusetts, NE). Carotenoids were eluted at 1 ml min^-1 ^with a gradient 95% A, 5% B for 12 min, then 5% B, 15-65% C from 12-30 min. Buffer A was 100% MeOH, Buffer B, 80% MeOH, 0.2% ammonium acetate and Buffer C tert-methyl butyl ether. Carotenoids were identified based on their retention time, visible and mass data [63,64], (Additional file 5), and were quantified at 450 nm as lutein equivalents.

### Sugar analysis

From roughly 50 mg of freeze dried powder, sugars were extracted twice with a total of 2 ml 80% ethanol by heating for 15 min at 80°C. The two extracts were combined, and an aliquot of 300 μl was dried over night at 50°C. The dried pellet was dissolved in 300 μl of water. Sucrose and glucose in the samples were estimated using Sigma kits (SCA20, GAHK20) as described by the manufacturer using 20 μl of the sample extract.

### RNA extraction and qPCR

RNA was extracted from freeze dried tuber samples using the CTAB (cetyltrimethylammonium bromide) method [65]. Fifty mg of dry powder was mixed with 1 ml of CTAB buffer (2% w/v CTAB, 1.4 M NaCl, 20 mM EDTA, 0.1 M Tris-HCl pH 8.0, 2% w/v PVP (K-30), 0.2% v/v β-mercaptoethanol) and vortexed for 3 min. Then 700 μl of acid phenol:chloroform:isoamyl alcohol (125:24:1) was added, vortexed for 2 min, incubated for 5 min at 65°C, and centrifuged for 10 min at 13,000 g. The supernatant was reextracted with 600 μl of chloroform:isoamyl alcohol (24:1). After adding 400 μl of 8 M LiCl, samples were incubated overnight at 4°C, then centrifuged for 10 min at 4°C. The pellet was dissolved in 300 μl ddH_2_O, and precipitated by adding 30 μl of 3 M sodium acetate (pH 5.2) and 750 μl of 95% ethanol, incubating at -80°C for 1 hr and centrifuging at 4°C for 20 min. RNA quantity was assessed by using a NanoDrop ND-1000 spectrophotometer (NanoDrop Technologies, Wilmington, DE) and quality was assessed by running 375 ng RNA on a 1% agarose gel. cDNA was synthesized using 2 μg total RNA, anchored oligo(dT) 20VN primers and M-MuLV reverse transcriptase (New England BioLabs).

Relative transcript abundance was analyzed by qPCR in a 12 μl reaction volume with 4 ng RNA equivalent cDNA, 400 nM gene-specific primers and 6 μl SYBR Green Mix (Roche, Mannheim, Germany). Amplification was 5 min preincubation at 95°C followed by 40 cycles of 10 sec denaturation at 95°C, 20 sec annealing at 60°C, and 20 sec extension at 72°C, using a LightCycler 480 (Roche). Three biological and two technical replicates were used for each determination. Relative expression was calculated by the ΔCT method [66] by normalizing the CT levels of target genes to the geometric mean of CT levels of two housekeeping genes, cytoplasmic ribosomal protein L2 and elongation factor 1-α(EF1-α). Specificity of amplification was assessed by dissociation curve analysis and running the PCR product on gel. The primer sequences are given in Additional file 6 and sequences for L2, EF1-α, PAL, C4H, HQT, CHS, F3H, DFR, ZDS, LCY-e, LUT1 are previously reported [27,53].

### PAL and FRAP assays

Soluble proteins were extracted from 50 mg freeze dried powder with 750 μl of cold 50 mMTris-Cl (pH 8.8) containing 1 mM EDTA, 1 mM PMSF, 5.7 mM β-mercaptoethanol, 1% insoluble PVPP, and 0.2% Triton X-100. Protein concentration was estimated using Bradford reagent with bovine serum albumin (BSA) as standard. The enzyme reaction was performed as described [67] with modifications. The reaction media (1 ml) consisted of 100 μl of the protein extract, 10 mM phenylalanine and 50 mM final concentration of sodium borate buffer (pH 8.8). After incubating the samples for 1 hr at 37°C, the reaction was stopped by adding 200 μl of 12% TCA, centrifuged for 10 min, and the A_290 _measured. Ferric Reducing Antioxidant Power (FRAP) assay was performed as reported [68] and the average of two experiments reported. Antioxidant values were calculated as trolox equivalents.

### Promoter analysis

Promoter sequences for phenylpropanoid genes were obtained from the Potato Genome Sequencing Consortium database [69]. Contig numbers are given in Additional file 6. Gene structure was analyzed using online software [70]. The 1500 bp upstream of ATG was analyzed using the PlantCARE website [71].

### Data analysis

Data analysis was performed in Microsoft excel and analysis of variance (ANOVA) was performed using SAS 9.2 Proc GLM. Means were obtained using LSMEANS with Tukey adjustments for the degrees of freedom and *P *< 0.05 default separation. Heatmaps were generated with Heatmapper Plus [72], using Pearson product moment correlation coefficients (r values) calculated using the means of metabolite concentrations or relative gene expression values.

## Abbreviations

4CL: 4-coumaroyl:CoA-ligase; A3'MT: anthocyanin 3'-methyltransferase; A3'5'MT: anthocyanin 3',5'-methyltransferase; ACT: anthocyanin acyltransferase; ANS: anthocyanin synthase; Ant: total anthocyanins; Anthera: antheraxanthin; ART: anthocyanin rhamnosyltransferase; Asc: ascorbate; BDCS: bis-dihydrocaffeoylspermine; BDCSD: bis-dihydrocaffeoylspermidine; C3H: p-coumarate 3-hydroxylase; C4CGA and C5CGA: chlorogenic acid isoform; C4H: cinnamate 4-hydroxylase; CA: caffeic acid; CAP: caffeoylputrescine; cCGA: crypto chlorogenic acid; CGA: chlorogenic acid; CHI: chalconeisomerase; CHS: chalcone synthase; CHY1: and 2: β carotene hydroxylase isoforms: Crt: total carotenoids; CRT iso: carotene isomerase; CyCRG: cyanidin 3-coumuryol rutinoside 5-glucoside; DeCRG: delphinidin 3-coumuryol rutinoside 5-glucoside; DFR: dihydroflavonol 4-reductase; F3H: flavanone 3-hydroxylase; F3'H: flavonoid 3' hydroxylase; F3'5'H: flavonoid 3'5' hydroxylase; FAQ1 and FAQ2: feruloylquinic acid isoforms; FLS: flavonol synthase; FRAP: ferric reducing antioxidant power; GA: glycoalkaloids; Glu: glucose; HCGQT: hydroxycinnamoylglucose:quinatehydroxycinnamoyl transferase; HCT: hydroxycinnamoyl Co A shikimate hydroxycinnamoyl transferase; HQT: hydroxyl-cinnamoyl CoA quinatehydroxycinnamoyl transferase; Kmp: kaempferol-3-O-rutinoside; LCY-b1 and b2: lycopene β cyclase isoforms; LCY-e: lycopene ε cyclase; LUT1: carotene hydroxylase; MaCRG: malvidin 3-coumuryol rutinoside 5-glucoside; Myr: myricetin-3-O-rutinoside; nCGA: neochlorogenic acid; Neo: neoxanthin; NXS: neoxanthin synthase; PAL: phenylalanine ammonia-lyase; PALe: PAL enzyme activity; PDS: phytoenedesaturase; PeCRG: peonidin 3-coumuryol rutinoside 5-glucoside; Phe: phenylalanine; Phn: total phenolics; PlCRG: pelargonidin 3-coumuryol rutinoside 5-glucoside; PSY1 and 2: phytoene synthase isoforms; PtCRG: petunidin 3-coumuryol rutinoside 5-glucoside; Ptd: petunidin derivative; Rut: quercetin-3-O-rutinoside; Suc: sucrose; TDCS: tris-dihydrocaffeoyl spermine; TDCSD: tris-dihydrocaffeoyl spermidine; Trp: tryptophan; Tyr: tyrosine; UF5GT: UDPG flavonoid 5-O-glucosyltransferase; UFGT: UDP glucose:flavonolglucosyl transferase; UGCT: UDP glucose:cinnamateglucosyl transferase; VDE: violaxanthin de-epoxidase; Viola: violaxanthin; ZDS: ζ -carotene desaturase; Z-ISO: ζ -carotene isomerase; Zea: zeaxanthin; ZEP: zeaxanthin epoxidase

## Authors' contributions

RSP carried out the LCMS, sugar, qRT-PCR analysis and contributed to the writing. DAN conceived the study, guided LCMS method development and analysis and wrote the manuscript. JCK and AP grew the Alaskan potatoes and designed the field component. SSP performed the antioxidant assays. All authors read and approved the final version of the manuscript.

## Supplementary Material

Additional file 1 **Table of phenolic compounds measured in tubers by LCMS**. Retention time (*R*t) and MS data of analyzed compounds is shown. Click here for file

Additional file 2 **Relative expression of five genes involved in primary metabolism**. DAHP, 3-deoxy-D-arabino-heptulosonate 7-phosphate synthase; PGK, phosphoglycerate kinase; AMY, alpha-amylase; SUSY, sucrose synthase; SSY, soluble starch synthase. The data represents the means ± SE of three biological replicates. Locations with same letter are not significantly different (p < 0.05). Click here for file

Additional file 3 **Carotenoid profiles in Alaska (A), Texas (B) and Florida (C) samples**. Major peaks are 1. neoxanthin, 2. violaxanthin, 3. antheraxanthin, 4. lutein, 5. zeaxanthin, 6. β-apo-caroten-8-ol internal standard. Click here for file

Additional file 4 **Correlation analysis of carotenoid transcript and metabolite levels in tubers**. Pearson correlation coefficients were calculated and used to generate a heatmap in which positive correlations are shown in red and negative correlations in blue. Phen, total phenolics; Anth, total anthocyanins; Cartn, total carotenoids; FRAP, antioxidant capacity. Additional abbreviations are listed in Figure 8 and Additional file 1. Click here for file

Additional file 5 **Table of HPLC data of tuber carotenoids**. Click here for file

Additional file 6 **Sequence information for primers**. Click here for file
