# Supplementary material for: Differential effects of environment on potato phenylpropanoid and carotenoid expression
Source: BMC Plant Biol. 2012 Mar 20;12:39. doi: 10.1186/1471-2229-12-39 (PMC3342224; doi:10.1186/1471-2229-12-39)
Supplement: Additional file 1 — Table of phenolic compounds measured in tubers by LCMS. Retention time (Rt) and MS data of analyzed compounds is shown. [file 1471-2229-12-39-S1.DOCX]

**Additional file 1.** Relative expression of five genes involved in primary metabolism. DAHP, 3-deoxy-D-arabino-heptulosonate 7-phosphate synthase; PGK, phosphoglycerate kinase; AMY, alpha-amylase; SUSY, sucrose synthase; SSY, soluble starch synthase.The data represents the means ± SE of three biological replicates. Locations with same letter are not significantly different (p< 0.05).
