# Supplementary material for: Differential effects of environment on potato phenylpropanoid and carotenoid expression
Source: BMC Plant Biol. 2012 Mar 20;12:39. doi: 10.1186/1471-2229-12-39 (PMC3342224; doi:10.1186/1471-2229-12-39)
Supplement: Additional file 2 — Relative expression of five genes involved in primary metabolism. DAHP, 3-deoxy-D-arabino-heptulosonate 7-phosphate synthase; PGK, phosphoglycerate kinase; AMY, alpha-amylase; SUSY, sucrose synthase; SSY, soluble starch synthase. The data represents the means ± SE of three biological replicates. Locations with same letter are not significantly different (p < 0.05). [file 1471-2229-12-39-S2.DOCX]

**Additional file 2.** Carotenoid profiles in Alaska (A), Texas (B) and Florida (C) samples. Major peaks are 1. neoxanthin, 2. violaxanthin, 3. antheraxanthin, 4. lutein, 5. zeaxanthin, 6. β-apo-caroten-8-ol internal standard.
