# Supplementary material for: Differential effects of environment on potato phenylpropanoid and carotenoid expression
Source: BMC Plant Biol. 2012 Mar 20;12:39. doi: 10.1186/1471-2229-12-39 (PMC3342224; doi:10.1186/1471-2229-12-39)
Supplement: Additional file 3 — Carotenoid profiles in Alaska (A), Texas (B) and Florida (C) samples. Major peaks are 1. neoxanthin, 2. violaxanthin, 3. antheraxanthin, 4. lutein, 5. zeaxanthin, 6. β-apo-caroten-8-ol internal standard. [file 1471-2229-12-39-S3.DOCX]

Additional file 3: Correlation analysis of carotenoid transcript and metabolite levels in tubers. Pearson correlation coefficients were calculated and used to generate a heatmap in which positive correlations are shown in red and negative correlations in blue. Phen, total phenolics; Anth, total anthocyanins; Cartn, total carotenoids; FRAP, antioxidant capacity. Additional abbreviations are listed in Figure 8 and Additional file 4.
