# Supplementary material for: Differential effects of environment on potato phenylpropanoid and carotenoid expression
Source: BMC Plant Biol. 2012 Mar 20;12:39. doi: 10.1186/1471-2229-12-39 (PMC3342224; doi:10.1186/1471-2229-12-39)
Supplement: Additional file 5 — Table of HPLC data of tuber carotenoids. [file 1471-2229-12-39-S5.DOCX]

Additional file 5. Sequence information for primers.
