# Supplementary material for: Differential effects of environment on potato phenylpropanoid and carotenoid expression
Source: BMC Plant Biol. 2012 Mar 20;12:39. doi: 10.1186/1471-2229-12-39 (PMC3342224; doi:10.1186/1471-2229-12-39)
Supplement: Additional file 6 — Sequence information for primers. [file 1471-2229-12-39-S6.DOCX]

**Additional file 6.** Carotenoids measured in tubers by HPLC.

| **RT** |  | **Compound** | **λ max (nm)** | | |
| --- | --- | --- | --- | --- | --- |
| 8.9 |  | Neoxanthin | 415 | 439 | 467 |
| 9.9 |  | Violaxanthin | 415 | 438 | 467 |
| 11.6 |  | cis-Neoxanthin | 412 | 435 | 463 |
| 16.4 |  | Antheraxanthin | 422 | 443 | 472 |
| 16.8 |  | Chlorophyll b |  |  | 468 |
| 17.7 |  | Lutein | 421 | 444 | 472 |
| 18.9 |  | Zeaxanthin | 424 | 449 | 478 |
| 20.7 |  | cis-α-carotene | 417 | 438 | 469 |
| 22.2 |  | 5,6-epoxy-α-carotene | 416 | 439 | 469 |
| 26.2 |  | β-Carotene | 424 | 450 | 477 |
